# Private dental insurance expenditure in Brazil

**DOI:** 10.11606/S1518-8787.2018052000340

**Published:** 2018-02-07

**Authors:** Andreia Morales Cascaes, Maria Beatriz Junqueira de Camargo, Eduardo Dickie de Castilhos, lexandre Emídio Ribeiro Silva, Aluísio J D Barros

**Affiliations:** I Universidade Federal de Pelotas. Faculdade de Odontologia. Departamento de Odontologia Social e Preventiva. Pelotas, RS, Brasil; II Universidade Federal de Pelotas. Faculdade de Medicina. Centro de Pesquisas Epidemiológicas. Pelotas, RS, Brasil

**Keywords:** Insurance, Dental, utilization, Insurance, Health, Prepaid Health Plans, Health Expenditures, Population Surveys, Seguro Odontológico, utilização, Seguro Saúde, Planos de Pré-Pagamento em Saúde, Gastos em Saúde, Inquéritos Demográficos

## Abstract

**OBJECTIVE:**

To quantify the household expenditure *per capita* and to estimate the percentage of Brazilian households that have spent with dental insurance.

**METHODS:**

We analyzed data from 55,970 households that participated in the research *Pesquisa de Orçamentos Familiares* in 2008–2009. We have analyzed the annual household expenditure *per capita* with dental insurance (business and private) according to the Brazilian states and the socioeconomic and demographic characteristics of the households (sex, age, race, and educational level of the head of the household, family income, and presence of an older adult in the household).

**RESULTS:**

Only 2.5% of Brazilian households have reported spending on dental insurance. The amount spent *per capita* amounted to R$5.10 on average, most of which consisted of private dental insurance (R$4.70). Among the characteristics of the household, higher educational level and income were associated with higher spending. São Paulo was the state with the highest household expenditure *per capita* (R$10.90) and with the highest prevalence of households with expenditures (4.6%), while Amazonas and Tocantins had the lowest values, in which both spent less than R$1.00 and had a prevalence of less than 0.1% of households, respectively.

**CONCLUSIONS:**

Only a small portion of the Brazilian households has dental insurance expenditure. The market for supplementary dentistry in oral health care covers a restricted portion of the Brazilian population.

## RESUMO

**OBJETIVO:**

Quantificar as despesas domiciliares *per capita* e estimar o percentual de domicílios brasileiros que gastaram com planos exclusivamente odontológicos.

**MÉTODOS:**

Foram analisados dados de 55.970 domicílios que participaram da Pesquisa de Orçamentos Familiares em 2008–2009. Os gastos domiciliares anuais *per capita* com planos exclusivamente odontológicos (empresarial e particular) foram analisados segundo os estados da federação e as características socioeconômicas e demográficas dos domicílios (sexo, idade, cor da pele e escolaridade do chefe do domicílio, renda familiar e presença de idoso no domicílio).

**RESULTADOS:**

Apenas 2,5% dos domicílios brasileiros relataram gastos com planos exclusivamente odontológicos. O valor *per capita* despendido somou em média R$5,10, sendo a maior parte composta por planos odontológicos particulares (R$4,70). Entre as caraterísticas do domicílio, maior escolaridade e renda estiveram associadas com maior gasto. São Paulo foi o estado com maior gasto domiciliar *per capita* (R$10,90) e maior prevalência de domicílios com dispêndios (4,6%), enquanto Amazonas e Tocantins apresentaram os menores valores, ambos com gasto inferior a R$1,00 e com menos de 0,1% de domicílios, respectivamente.

**CONCLUSÕES:**

Apenas uma pequena parcela dos domicílios brasileiros desembolsa com planos exclusivamente odontológicos. O mercado de odontologia suplementar na assistência em saúde bucal abrange uma restrita parcela da população brasileira.

## INTRODUCTION

Dentistry in Brazil, historically, has always had its practice centered on private clinics^9^. However, the increase in the number of professionals available in the market, the decrease in the direct disbursement of patients to pay for treatments, and the unfair use of private services have led to the crisis of the liberal model^5^ ^,^ ^15^. Faced with this situation, dental insurance, in its various modalities, has been shown as an option in the current labor market^15^.

Following the publication of Law 9,656 in 1998, which provides for private health plans and insurances and establishes the main health regulatory frameworks in Brazil^a^, and the creation of the National Supplementary Health Agency (ANS) in 2000^b^, we can observe a large increase in the number of beneficiaries by providers of dental services. Beneficiaries include links to health insurance; thus, an individual may have more than one health insurance and therefore more than one contractual link^1^. The percentage of beneficiaries increased from 2.6% in December 2000 to 21.7% in March 2016^c^. In March 2016, coverage rates of dental insurance in Brazil ranged from 1.4% in Roraima to 31% in the Federal District^c^. In this same period, 351 dental providers had active registration in the ANS, being 241 of them group providers and 110 cooperatives, representing, respectively, 18.4% and 8.4% of the active providers in the supplementary health sector in Brazil^c^.

The observed increase in the number of beneficiaries of dental insurance can be attributed to changes in the profile of dentistry, business strategies, privatizations, and difficulties in accessing oral health services, as well as tax incentives and deductions in personal and business income tax^11^ ^,^ ^15^. Together with the expansion of this market, the supply of dental and specialized services significantly increased, driven by the promulgation of the National Oral Health Policy in 2004^d^. The advancement of dentistry in the public sector, although great, has not yet been enough to absorb all the existing demand. Despite the growth of the supplementary dentistry market in Brazil, the total number of beneficiaries (21.7 million) is still far from the total of 48.9 million of persons with health care insurance with or without dentistry^c^.

National data on direct disbursement with health care and health insurance can be obtained from the research *Pesquisa de Orçamentos Familiares* (POF) of the Brazilian Institute of Geography and Statistics (IBGE), complementing the information provided by the ANS. Reis et al.^13^, using data from the 1987 and 1996 POF, have shown that, among families with a monthly average income greater than 30 minimum wages, the proportional percentage of health insurance expenditure went from 54.3% in 1987 to 40.4% in 1996, which indicates a greater acquisition of the insurance by lower income families. Garcia et al.^7^ have used data from the 2002–2003 and 2008–2009 POF to investigate the proportion of households that spent on health insurance (including dental insurance). The authors have found that the proportion remained stable, at approximately 24%. However, the average value of expenditure among households increased from R$154.35 to R$183.97^7^.

In the last three decades, mainly after government regulation, the availability of basic information on providers and the number and distribution of beneficiaries and health insurance improved^1^. However, the market for dental insurance is still little known and rarely considered in the planning of the offer of dental services in Brazil. The scientific production in the field of epidemiology and collective oral health has prioritized the evaluation of policies and services of state order. It is relevant to analyze available data, contributing with the evaluation of the supplementary health market in dentistry and with the formulation of public policies and planning of health services.

We identified no studies in the literature that specifically investigate the amount and number of households that have spent on dental health insurance in Brazil. To this end, we aimed to quantify the expenditures and the percentage of Brazilian households that have spent on dental insurance using data from the 2008–2009 POF, and we also aimed to describe them according to the socioeconomic and demographic characteristics of the households and Brazilian states.

## METHODS

We analyzed data from 55,970 households that participated in the research *Pesquisa de Orçamentos Familiares* (POF) carried out in 2008–2009. The POF is a national research carried out by the IBGE to characterize the expenditures and consumption of Brazilian families according to their income classes, providing detailed information on households, families, and persons, as well as consumption habits, expenses, and income of the families^e^.

The POF used a complex sampling plan, in which the census tracts were first selected, followed by the households. Households were distributed equally in the four quarters of the research, taking into account possible changes in expenditure throughout the year and ensuring the representation of the geographic and socioeconomic strata^e^.

The sample of the POF allows us to infer for the five macroregions (North, Northeast, South, Southeast, and Midwest), the 26 federation units, the nine metropolitan regions, and the state capitals in relation to the urban zone. The five Brazilian macroregions can be divided between rural and urban areas. Two out of the 4,696 sectors presented all interviews as not carried out and therefore were excluded from the sample analyzed in this study.

### Socioeconomic and Demographic Variables

The following variables were included in the analysis: states of the country, sex of the head of the household (male, female), educational level of head of household in years of study (0–4 years, 5–8 years, 9–11 years, ≥ 12 years), race of the head of the household (white, brown, black, yellow, indigenous), age of the head of household in full years (≤ 29 years, 30–39 years, 40–49 years, 50–59 years, ≥ 60 years), quintiles of the monthly household income *per capita*, and presence of an older adult aged 60 years or more in the household. We considered the monthly household income *per capita* and the educational level of the head of the household as indicators of socioeconomic status. In households with more than one head, we used the information of the oldest head as reference for the analysis.

### Dental Insurance Expenditure

We analyzed the total amount and the average annual *per capita* value spent with dental insurance (business and private). The recall period for the expenditure was 90 days. To estimate household expenditures *per capita*, we divided the total household expenditure by the number of residents in that household.

We considered households as units of analysis, as the decision to acquire a dental insurance is usually made by the head of the family who buys family insurance or makes the payment of the insurance to other family members.

### Analysis of the Data

The databases of the POF are in the public domain and were obtained from the IBGE website. We analyzed the data using the statistical package Stata/IC, version 14.0, considering the sampling design of the POF and the expansion factors provided by the IBGE. The values were deflated by the POF using as reference the date of January 15, 2009. The value of the minimum wage in the same period corresponded to R$415.00. The exchange rate of the dollar at that date was R$2.38, equivalent to U$1.00.

We estimated the total health expenditures of families in order to know the total percentage of the dentistry expenditure. The dental expenditure block was then detailed to investigate how much the dental insurance expenditure represents in relation to the total of this block. Subsequently, we analyzed dental insurance expenditures according to the demographic and socioeconomic characteristics of Brazilian households and states. We analyzed the expenditures on basic and specialized dental appointments and treatments, as categorized previously in the literature^4^, according to the dental insurance expenditure.

To facilitate the interpretation and presentation of the results, we chose to divide the absolute numbers of households by 1,000. Finally, we performed an adjusted analysis of the association between dental insurance expenditures and the demographic and socioeconomic characteristics of the households. Mean ratios and 95% confidence intervals were estimated from crude and adjusted Poisson regressions. We included, in the final adjustment, the variables whose p-value in the crude analysis was < 0.2. The final adjustment model considered as variables associated with dental insurance expenditures those that presented value of p < 0 .05.

## RESULTS

According to the 2008–2009 POF, the average annual health care expenditure in Brazil amounted to R$1,849.74, which is equivalent to 5.9% of the total household expenditure. The total annual household expenditure *per capita* on health care was R$543.70, of which R$42.19 were spent on dental care (dental insurance, appointments, and treatments). Of the total dental care expenditure, R$5.10 were related to dental insurance, and most consisted of expenses with private dental insurance (R$4.07). Only 2.5% of Brazilian households reported spending on dental insurance.

Table 1 shows the distribution of the households researched and annual household expenditure *per capita* on dental insurance according to the characteristics of the household. Regarding the characteristics of the head of the household, approximately 70.0% are male, 23.8% are aged 60 years or more, and 40.0% have between zero and four years of educational level. There was no difference in the average expenditures *per capita* among the heads of household according to sex, age, and race of the head and the presence of an older adult in the household. However, dental insurance expenditure increases as educational level increases. Income follows the same trend as educational level.

**Table 1:** Distribution of households researched and annual household expenditure *per capita* with dental insurance, according to characteristics of the household. *Pesquisa de Orçamentos Familiares*, Brazil, 2008–2009.

Characteristics of the families	Distribution		Dental insurance expenditure
	
	n*	%	Average in Real (95%CI)
Sex of the head			p = 0.067
Male	39,747	68.9	4.23 (3.41–5.04)
Female	17,945	31.1	7.03 (3.39–10.67)
Age of the head (years)			p = 0.545
≤ 29	7,275	12.6	3.39 (1.53–5.25)
30–39	12,248	21.1	5.58 (3.62–7.54)
40–49	13,480	23.3	5.85 (3.79–7.91)
50–59	10,930	18.9	5.52 (4.05–6.99)
≥ 60	13,758	23.8	4.50 (0.18–8.81)
Educational level of the head (years)			p < 0.001
0–4	23,554	40.8	1.30 (0.80–1.79)
5–8	12,725	22.1	3.17 (2.25–4.08)
9–11	13,302	23.1	6.75 (5.04–8.46)
≥ 12	8,111	14.1	16.47 (8.29–24.64)
Race of the head			p = 0.106
White	28,394	49.4	5.40 (4.24–6.55)
Brown	23,383	40.6	4.38 (1.81–6.93)
Black	5,128	8.9	6.18 (1.36–10.99)
Yellow	347	0.6	14.52 (-3.11–32.11)
Indigenous	259	0.5	1.81 (0.02–3.62)
Quintile of household income *per capita*			p < 0.001
1 (average R$158.47)	11,540	20.0	0.53 (0.25–0.81)
2 (average R$347.54)	11,540	20.0	1.29 (0.90–1.68)
3 (average R$575.38)	11,535	20.0	2.96 (2.03–3.89)
4 (average R$963.95)	11,538	20.0	6.00 (4.35–7.65)
5 (average R$3,209.17)	11,538	20.0	14.71 (8.74–20.68)
Presence of older adult aged 60 years or more in the household			p = 0.647
No	41,730	72.3	5.37 (4.30–6.46)
Yes	15,961	27.7	4.38 (0.65–8.10)

* Number of households expanded and divided by 1,000 (values adjusted by the sample weight).

The adjusted analysis of the association between dental insurance expenditures and characteristics of the household is described in Table 2. We included only the sex of the head, educational level of the head, and family income in the adjusted analyzes. Only educational level and income remained associated. Among the richest, average expenditure was 13 times higher, after adjustment for educational level and sex of the head of household (Table 2).

**Table 2:** Crude and adjusted Poisson regressions between annual household expenditure *per capita* with dental insurance and characteristics of the household. *Pesquisa de Orçamentos Familiares*, Brazil, 2008–2009.

Characteristics of the families	AR_c_ (95%CI)	AR_a_ (95%CI)
Sex of the head	p = 0.067	p = 0.058
Male	1.00	1.00
Female	1.66 (0.96–2.87)	1.65 (0.98–2.78)
Age of the head (years)	p = 0.545	*
≤ 29	1.00	
30–39	1.64 (0.85–3.16)	
40–49	1.73 (0.90–3.30)	
50–59	1.62 (0.88–3.00)	
≥ 60 or more	1.32 (0.44–4.01)	
Educational level of the head (years)	p < 0.001	p < 0.001
0–4	1.00	1.00
5–8	2.44 (1.51–3.95)	2.13 (1.31–3.47)
9–11	5.21 (3.34–8.12)	3.48 (2.21–5.49)
≥ 12 or more	12.70 (6.80–23.74)	4.94 (2.79–8.75)
Race of the head	p = 0.106	*
White	1.00	
Brown	0.81 (0.44–1.51)	
Black	1.15 (0.52–2.54)	
Yellow	2.70 (0.78–9.19)	
Indigenous	0.34 (0.12–0.93)	
Quintile of household income *per capita*	p < 0.001	p < 0.001
1 (average R$158.47)	1.00	1.00
2 (average R$347.54)	2.43 (1.33–4.42)	2.11 (1.16–3.85)
3 (average R$ 575.38)	5.58 (3.05–10.21)	4.54 (2.46–8.36)
4 (average R$963.95)	11.32 (6.28–20.40)	7.72 (4.25–14.00)
5 (average R$3,209.17)	27.75 (14.39–53.55)	13.16 (7.14–24.25)
Presence of older adult aged 60 years or more in the household	p = 0.647	*
No	1.00	
Yes	0.81 (0.34–19.6)	

AR_c_: crude average ratio; AR_a_: adjusted average ratio

* Variables with p value > 0.20 were not considered in the adjusted model.

Table 3 analyzes the expenditures on dental appointments and treatments according to dental insurance disbursement. The results show that households that have insurance disbursement spent significantly more with dental appointments and specialized procedures. On the other hand, dental insurance disbursement did not change expenditure on basic procedures.

**Table 3:** Average annual expenditure *per capita* with dental appointments and basic and specialized dental procedures according to the disbursement of dental insurance. *Pesquisa de Orçamentos Familiares*, Brazil, 2008–2009.

Annual expenditure *per capita* (in R$)	Disbursement with dental procedures		p

	No	Yes	
Dental appointments	1.28	2.15	0.026
Specialized dental procedures	33.7	87.5	0.025
Basic dental procedures	0.72	0.58	0.687

**Figure 1:**
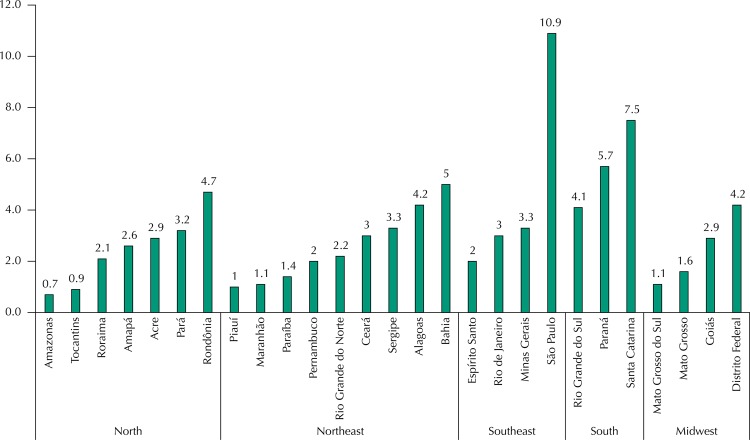
Household expenditure *per capita* (in Real) with dental insurance, according to the Brazilian states. *Pesquisa de Orçamentos Familiares*, Brazil, 2008–2009.

Figure 1 shows the household expenditure *per capita* with dental insurance according to the states of the federation. The states of São Paulo (R$10.90), Santa Catarina (R$7.50), and Paraná (R$5.70) were the ones that had the highest average expenditure. Among those that spent less, we have Amazonas (R$0.70), Tocantins (R$0.90), and Piauí (R$1.00).

**Figure 2:**
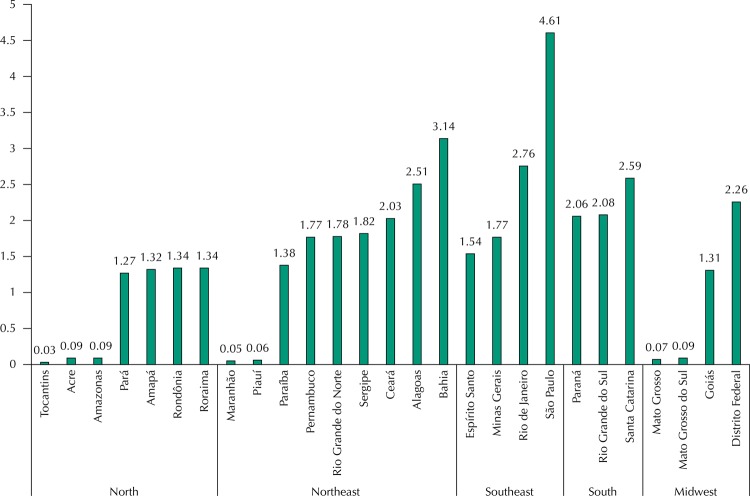
Percentage of households that spent (*per capita*) with dental insurance, according to the Brazilian states. *Pesquisa de Orçamentos Familiares*, Brazil, 2008–2009.

Figure 2 presents the prevalence of households that reported dental insurance expenditure. São Paulo (4.6%), Bahia (3.1%), and Rio de Janeiro (2.8%) were the three states with the highest prevalence of households with expenditures. Among the states with the lowest proportion of households with these expenditures we have Tocantins, Maranhão, and Piauí, all with less than 0.1%.

## DISCUSSION

To our knowledge, this study is the first one, at the national level, that quantifies in detail the expenditures and the percentage of Brazilian households that reported disbursements with dental insurance. Less than 1% of the total annual health expenditures (R$1,849.74) is due to the use of dental insurance. In relation to the total amount spent on dental care (R$42.19), insurance expenditure is approximately 12%, and the proportion of households with this type of expenditure was 2.5% in the country.

The distinctions between medical and dental care imply differences in the structure of the insurances and, consequently, in their acquisition. As discussed by Covre and Alves^f^, consumers choose to acquire medical-hospital insurance in order to protect themselves financially against a risk of illness that, in most cases, is uncertain. The risks for oral health are reasonably predictable and, in general, limited to the consequences of the two most prevalent oral diseases, caries and periodontal disease. With the exception of trauma, accidents, or pain of dental origin, the need for dental treatment is rarely a life-threatening emergency. In this sense, individuals can plan when they wish to undergo a treatment. Therefore, consumers acquire dental insurance in order to finance the investment that they want to make on their teeth. After the first dental appointment, the individual receives a forecast of how many appointments will be required to complete the treatment. Following adherence to the dental insurance, we can observe a peak in the use, with a high frequency of low-cost events and subsequent stabilization trend, reducing costs and losses over time. In addition, the consumer has the possibility of having a dental insurance, undergoing the treatment, and canceling it. For these reasons, there is no direct relationship between increasing age and level of use of dental insurance, the same finding as in our study in relation to the expenditure pattern. As a result of these particularities, the frequency of Brazilian households that spend with dental insurance is still timid when compared to the medical area – 24% with medical care insurance^7^ *versus* 2.5% with dental insurance, according to data from the 2008–2009 POF.

Higher educational level and higher family income *per capita* were associated with higher dental insurance expenditure. The same association is highlighted in several studies on health care expenditures^2^ ^,^ ^3^ ^,^ ^6^ ^,^ ^7^. Purchasing power is determinant when buying health insurance or it may be representing individuals who have more qualified jobs that offer dental insurance as a benefit. When comparing the differences in coverage of dental insurance provided by the ANS and the percentage of households that disbursed with this type of service, we can suggest that most users have the benefit granted by the employer. In 2016, 73% of the dental insurance was the collective corporate type and 8% were the collective by adhesion type, which indicates that this benefit is subsidized by the employer, fully or partially^c^. Among the households that reported spending on dental insurance, more than 90% disbursed with private insurance; thus, only a small portion adds resources to the payment of insurance granted by companies. Dentistry has been established among the benefits offered to individuals inserted in the formal labor market, but who do not constitute the economic elite, with the prerogative to reduce absenteeism, increase productivity, and improve self-esteem and quality of life^11^.

Even if the intention when acquiring health insurance is to reduce direct disbursements with treatments, this does not seem to occur. Barros et al.^2^ have used data from the 2002–2003 POF to show that the presence of insurance was not able to prevent out-of-pocket health care expenditure. In addition to the double coverage of health services, the supplementary nature of the private insurance sector in Brazil introduces problems related to access and use of services and to the pocket of consumer citizens. This segmentation not only penalizes poorer individuals, but it also causes them to spend twice as much on health care^2^. Private insurance holders continue to use public services and are often favored in referral to the SUS for procedures not covered in the clause of the insurance, contributing to the increase in health inequities^14^.

In the case of dentistry, the presence of insurance does not also mean exemption from extra expenses, at least with specialized appointments and procedures, according to the data analyzed. The role of procedures included is usually limited to procedures of low complexity and lower cost. When it includes costly (e.g., specialized) procedures, the beneficiary still faces, in many situations, the refusal of dental surgeons to cover such procedure, since the value passed on by the providers is considered insufficient by the professionals^5^. The greater dental appointment expenditure for those who already disburse with insurance may be due to the requirement of co-participation imposed by the established contracts. As discussed earlier, the acquisition of dental insurance is generally linked to the search for the facilitation of the payments of specialized and expensive procedures, and not its total exemption. As with health insurance, the duplication of the expenditure for users of dental services with private insurance is notorious.

The study on the health expenditures of Brazilian families, carried out by Menezes et al.^g^ from data of the 2002–2003 POF, has identified that disbursement with drugs accounts for most of the expenditures among the poorest families (79.4% of the total health expenditure), and the highest disbursement among the richest is due to health insurance (82.8%). In this study, we observed a similar result, as the highest income quintile had dental insurance expenditure that was thirteen times higher when compared to the poorest quintile. In addition, the search for a medical-hospital health insurance in the groups with better socioeconomic conditions tends to be accompanied by the search for additional dental insurance^h^.

A higher educational level may result in greater concern about oral health and awareness of the importance of having access to dental services. In addition to the socioeconomic determinants studied, the purchase of health services and insurance is influenced by a complex range of factors, such as perceived need for treatment, psychosocial issues, social security, availability of public services, and epidemiological and sociocultural context, which may be also related to the purchase pattern for dental insurance^16^.

The hypothesis that households with female and older heads would present higher dental insurance expenditure was not confirmed. Analyses of the National Health Survey, carried out in 2013, reveal that women do not only use dental services more^i^, but they report having more need to consult with the dentist^10^. Data from the POF indicate increased dental insurance expenditure in households headed by women, although the association was not significant. In terms of age, the older the person, the greater the normative needs for dental treatment; however, the use of services^i^ and the perceived need to go to the dentist are inversely proportional to age. The high prevalence of edentulism and users of complete dentures in the Brazilian population are factors that contribute with the low regular use of dental services by older adults compared to younger ones, since many believe that the absence of natural teeth requires no dental care^8^. Another unconfirmed hypothesis is that households with white heads would spend more in relation to the other races; however, the direct disbursement with dental appointments, treatments, and insurance does not differ in relation to race in the POF^4^.

The states with the highest expenditures *per capita* were São Paulo, Santa Catarina, and Paraná. The coverage of health care insurance is higher among residents of urban areas, more populous municipalities, and more industrialized regions of the states, with higher income and supply of formal employment and health services, a situation found in the South and Southeast regions^1^ ^,^ ^12^. The pattern of out-of-pocket dental insurance expenditure seems to indicate the same rationale as the coverage of health care insurance. However, considering the low coverage of dental insurance, private services paid by users are still the main source of dental care in Brazil, and the Brazilian Unified Health System (SUS) occupies the second position^10^. The strengthening of dentistry as a public health policy accessible to the immense majority of Brazilians who depend on it remains a major challenge^5^.

The fact that the possession of dental insurance is concentrated on the most privileged socioeconomic strata and on the federated states with better economic development contributes to increase the inequities in the access and use of health services of the Brazilian population and in the development of private services that the population has greater difficulty of access^7^, as is the case with specialized dental services. The market for private dental insurance is also unequal in terms of coverage, with segmentation of products according to consumers, as well as various forms of structuring and financing. Although there are more than three hundred dental insurance providers, 74 of them concentrate 91% of the beneficiaries^c^.

In Brazil, there is a stagnation of the private sector of dentistry, with a concomitant expansion of the supplementary and dental service segment in the SUS. As in the medical field, dentistry in Brazil faces two movements: the first one is related to the strengthening of the insertion of oral health in the public policy scenario and the second one is directed to the growth of the supply of services in the supplementary sector^h^. Unlike the SUS, the Brazilian dental care supplement model focuses on the execution of curative and restorative procedures, with a predominance of hard and soft-hard technologies, becoming a model of high cost and low effectiveness^5^. The concepts of reception and bonding are not considered by dental surgeons, as well as the social, environmental, and behavioral determinants of the oral health-disease process^h^. The production of care is at the mercy of the contractual limits and the regulation practiced by the providers.

In order to adapt the supplementary care model to the principles of the SUS, the ANS has established rules in the relationships between providers, products, and beneficiaries^h^. After 2001, the number of cancellations of registrations of providers has increased, which may be associated with these new changes. Despite the advances, the ANS finds it difficult to fulfill its objectives, as it operates in a market that has been operating for almost 40 years and has expanded disorderly, with several insurance and providers that meet a wide variety of market interests and niches^1^.

The POF is carried out using a careful planning process, critical review of instruments, and primary data collection, which generate a valuable source of information on the budget of Brazilian families. However, this study is not free of limitations. One of them refers to the type of information, which is self-reported and which may have led to expenditure amounts that do not correspond to the amount actually spent. The 90-day reminder used to evaluate insurance expenditure helps reduce information bias, but it limits the collection of data on families that have spent on these insurances over a period of one year. The POF has information on individual possession of health insurance but not on dental insurance. The amounts reported refer to the direct expenditure of the families with the dental insurance, and we cannot evaluate the portion paid by companies to their employees, and we also cannot evaluate if the amount paid represents some percentage of the benefit granted by the companies. In this sense, we cannot estimate the coverage of individual ownership of dental insurance using the POF. The estimated coverage refers to the percentage of households with direct disbursement to purchase these insurances. Therefore, the percentage values found in the POF are not comparable to those of the ANS, which informs the national coverage of dental insurance, regardless of the type of disbursement. In addition, the coverage calculations presented by the ANS are an approximation, even after applying a correction factor, as the Beneficiary Information System registers the number of insurances, and this number is a little higher than the number of individuals^1^. Despite the mentioned limitations, this study provides additional knowledge about the magnitude and scope of the direct disbursements of Brazilian families with dental insurance.

This study showed that a small portion of the Brazilian households disbursed with dental insurance. The value *per capita* of this type of expenditure was, on average, R$5.10. Higher family income, higher educational level of the head, and households belonging to states with better socioeconomic levels had higher values *per capita* and higher prevalence of households with dental insurance expenditures. This study provides elements that allow a better understanding of the supplementary dentistry market in Brazil and complements information provided by the ANS. In a country with approximately 200 million inhabitants, and given the inequalities pointed out, the limitations of the supplementary dentistry market in the oral health care of Brazilians are evident. The continuity and qualification of the policy *Brasil Sorridente* should be reinforced, as well as the elaboration of proposals that contribute with the adequacy of the supplementary market to the principles and guidelines of the SUS.

## Footnotes

a Brasil. Lei número 9.656, de 3 de junho de 1998. Dispõe sobre os planos e seguros privados de assistência à saúde. *Diario Oficial Uniao*. 4 jun 1998. 29 jun 2000.

b Brasil. Lei número 9.961, de 28 de janeiro de 2000. Cria a Agência Nacional de Saúde Suplementar – ANS e dá outras providências. *Diario Oficial Uniao*. 29 jun 2000.

c Agência Nacional de Saúde Suplementar (BR). Beneficiários, operadoras e planos. *Cad Inf Saude Supl*. 2016 [cited 2017 Oct 19];10(1):1-65. Available from: http://www.ans.gov.br/images/stories/Materiais_para_pesquisa/Perfil_setor/Caderno_informacao_saude_suplementar/caderno_JUNHO_2016_total.pdf

d Ministério da Saúde (BR), Secretaria de Atenção à Saúde, Departamento de Atenção Básica, Coordenação Nacional de Saúde Bucal. Diretrizes da Política Nacional de Saúde Bucal. Brasília (DF); 2004. Available from: http://bvsms.saude.gov.br/bvs/publicacoes/politica_nacional_brasil_sorridente.pdf

e Instituto Brasileiro de Geografia e Estatística. Pesquisa de Orçamentos Familiares 2008-2009. Perfil das despesas no Brasil: indicadores selecionados. Rio de Janeiro: IBGE; 2012.

f Covre E, Alves SL. Planos odontológicos: uma abordagem econômica no contexto regulatório. Rio de Janeiro: Agência Nacional de Saúde Suplementar; 2002 [cited 2017 Oct 19]. (Série Regulação em Saúde, v.2). Available from: http://www.ans.gov.br/images/stories/Materiais_para_pesquisa/Materiais_por_assunto/ProdEditorialANS_Serie_regulacao_e_saude_Vol_2.pdf

g Menezes T, Campolina B, Silveira FG ,Servo LM, Piola S. O gasto e a demanda das famílias em saúde: uma análise a partir da POF de 2002- 2003. In: Silveira FG, Servo LM, Menezes T, Piola S, organizadores. Gasto e consumo das famílias brasileiras contemporâneas. Brasília (DF): Instituto de Pesquisa e Economia Aplicada; 2006 [cited 2017 Oct 19]. p.313-44. Available from: http://www.ipea.gov.br/portal/images/stories/PDFs/livros/livros/160104_gasto_e_consumo_familiar_vol1_ipea_2007_web.pdf

h Agência Nacional de Saúde Suplementar (BR). Planos odontológicos: evolução, desafios e perspectivas para a regulação da saúde suplementar. Rio de Janeiro: ANS; 2009 [cited 2017 Oct 20]. Available from: http://bvsms.saude.gov.br/bvs/publicacoes/planos_odontologicos.pdf

i Instituto Brasileiro de Geografia e Estatística. Pesquisa Nacional de Saúde 2013: acesso e utilização dos serviços de saúde, acidentes e violências. Brasil, Grandes Regiões e Unidades da Federação. Rio de Janeiro: IBGE; 2015.
